# Burnout and job stress of anesthesiologists in the tertiary class A hospitals in Northwest China: A cross-sectional design

**DOI:** 10.3389/fmed.2023.1140552

**Published:** 2023-04-11

**Authors:** Guang Yang, Lin-yuan Pan, Xiao-li Fu, Zhong Qing, Bu-huai Dong, Jiu-min Ye

**Affiliations:** ^1^Department of Anesthesiology, Xi'an Honghui Hospital Affiliated to Xi'an Jiaotong University, Xi'An, China; ^2^Department of Orthopaedics, Xiangya Hospital, Central South University, Changsha, Hunan, China; ^3^Department of Anesthesiology, Northwest Women's and Children's Hospital, Xi'An, China; ^4^Department of Orthopaedics, Xi'an Honghui Hospital Affiliated to Xi'an Jiaotong University, Xi'An, China

**Keywords:** anesthesiologist, burnout, job stress, questionnaire, tertiary class A hospital

## Abstract

**Purpose:**

Our purpose was to assess job stress and burnout among anesthesiologists in the tertiary class A hospitals in Northwest China, analyze the possible causes and adverse consequences of increased job stress and burnout of anesthesiologists in this region, and put forward suggestions in combination with the current national policies.

**Methods:**

We sent 500 electronic questionnaires to all anesthesiologists practicing in the tertiary class A hospitals in Northwest China from 1960 to 2017 on April 2020. A total of 336 (67.2%) questionnaires were returned and could be used for analysis. Burnout and job stress were assessed by using the modified Maslach Burnout Inventory—Human Services Survey and Chinese Perceived Stress Scale, respectively.

**Results:**

First, as for emotional exhaustion, the situations of anesthesiologists with different working years and workloads are different with statistical significance (*P* < 0.05). Second, as for depersonalization, the situations of anesthesiologists with different ages, professional titles, working years, physical health status, and workload are different (*P* < 0.05). Third, as for personal accomplishment, the situations of anesthesiologists with different physical health status are different (*P* < 0.05). Finally, the regression results showed that the longer the fatigue working years and the worse the physical health of anesthesiologists in Northwest China, the more likely these two factors were to cause burnout (*P* < 0.05), as for job stress, there was a negative correlation between job stress and physical health status (*P* < 0.05).

**Conclusion:**

Burnout and high job pressure are common among anesthesiologists in tertiary class A hospitals in Northwest China. We should focus on the allocation of labor intensity, pay attention to the physical and mental health of employees, establish targeted incentive mechanism, and improve the system of promotion and income rises for grassroots doctors. This may be not only conducive to the quality of medical care for patients but also conducive to the development of anesthesiology in China.

**Trial registration:**

Identifier: ChiCTR2000031316.

## Introduction

Burnout is a comprehensive symptom of physical and mental exhaustion and energy exhaustion, which includes emotional exhaustion, depersonalization, and reduced personal accomplishment (1). Job pressure refers to the pressure on people due to excessive workload, change of post, and excessive work responsibility (2).

An Italian study in 2020 assessed burnout among anesthesiologists and intensivists and showed that 79.9% (686) exhibited moderate levels of burnout (3). A cross-sectional study in Kosovo surveyed 154 anesthesiologists and anesthesia technicians in Kosovo and found that approximately one-third of Kosovar anesthesiologists and anesthesia technicians exhibited high levels of burnout (4). A U.S. study included 3,898 members of the American Society of Anesthesiologists and showed that 59.2% of the study subjects were at high risk of burnout (5). Numerous similar studies have shown a high risk of burnout in the group of anesthesiologists. As the world's most populous country, medical resources are relatively more scarce in China.

In China, the number of anesthesiologists per 10,000 population is only 0.4, far lower than the number of anesthesiologists per 10,000 population in developed countries in Europe and America (6). With the aging of the population in our country and increasing serious perioperative complications, the anesthesia risk is also increasing day by day. At the same time, the shortage of anesthesiologists and the uneven distribution of medical resources lead to increased job stress and burnout of anesthesiologists, which will not only affect the physical and mental health of anesthesiologists but also bring hidden dangers to the safety and quality of anesthesia (7).

Some studies have shown that the level of burnout in other occupations is related to the knowledge gap, for example, working sailors (8) and nurses in the operating room (9). There was also a knowledge gap in the level of burnout among frontline physicians in Bangladesh during the COVID-19 pandemic (10).

In Northwest China, the economy is relatively backward, the distribution of medical resources is unbalanced, and the level of medical and health services lags behind other regions (11). Therefore, to fully mobilize the enthusiasm of the medical staff, improve the level of medical services, and fully understand both the advantages and disadvantages of medical resources in this region is important for promoting the development of medical services. Thus, in this study, 500 anesthesiologists' job stress and burnout were investigated and analyzed in order to provide a reference for instructing anesthesiologists to relieve the pressure and improve the level of burnout.

## Methods

The Research Ethics Committee of Xi'an Honghui Hospital affiliated with Xi'an Jiaotong University (Chairperson: Peng Xu) approved the study on 9 April 2020, and written informed consent from patients was obtained.

This article adheres to the applicable Enhancing the QUAlity and Transparency of Health Research (EQUATOR) guidelines.

Using a random sampling method, an anonymous cross-sectional design was conducted among anesthesiologists in the tertiary class A hospitals in Northwest China. For sample size estimation, a pilot study of 167 cases before the survey indicated a burnout rate of 48%. We assumed that a 5% error was acceptable and then the confidence level was set at 95%. By considering the response rate, the sample size was 308. Based on this, a total of 500 questionnaires were sent out, of which 408 were recovered and 336 were effective. Thus, among the returned questionnaires, the effective rate was 82.4%.

### First section

The first section of the questionnaire included 11 questions designed to capture demographic, social, and work characteristics of the anesthesiologists, such as gender, age, educational background, professional title, working years, family economic status, physical health status, type of hospital, proportion of anesthesiologists in your department to the operating room, ratio of anesthesiologists to surgeons, and operation volume in recent 2 years.

### Second section

Burnout: The modified Maslach Burnout Inventory—Human Service Survey (MBI-HSS) (12) was used to assess burnout. The modified Maslach Burnout Inventory—Human Service Survey is widely used to assess burnout in healthcare workers. Research has shown that the Maslach Burnout Inventory—Human Service Survey has acceptable validity and reliability in measuring nurse burnout and can help healthcare administrators provide interventions to reduce nurse burnout (13). The measurement invariance of the MBI-HSS in gender and occupation was also confirmed in a Vietnamese study (14). The 16-item MBI is a measure of burnout and is subdivided into three subscales as follows: (a) Emotional exhaustion (six items): This subscale refers to a lack of emotional resources, that is, the feeling of having given everything and having nothing left to give. (b) Lack of personal accomplishment (six items): This subscale assesses feelings of doubt about one's ability to perform tasks and lack of successful achievement in one's work with people. (c) Depersonalization (four items): This subscale measures an unfeeling and impersonal response toward recipients of one's service, care, treatment, or instruction. The scoring method of four points was used, never so scored 0 points, rarely so scored 1 point, sometimes so scored 2 points, and often so scored 3 points. In the subscale of “emotional exhaustion”, values ≥19 are considered to indicate burnout; in the subscale of “personal accomplishment”, values ≤ 2.5 are considered to indicate burnout; and in the “depersonalization” subscale, values ≥17 are considered to indicate burnout.

### Third section

Job stress: The work stress of anesthesiologists was evaluated by the Chinese Perceived Stress Scale (CPSS) (15). The CPSS is a reliable and valid instrument for patients with common mental disorders with different employment status, exhibiting a stable two-factor structure with satisfactory internal consistency and construct validity (16, 17).

There were 14 items in CPSS, which had never scored 0, rarely scored 0.5, sometimes scored 1, and often scored 1.5. It was conceived to assess how many life situations are appraised as stressful, that is, how unpredictable and overloaded respondents find their lives. Higher scores indicate higher levels of job stress. At the end of the questionnaire, an open question was included for respondents to identify the main job stress factors encountered in their daily lives. The seven most frequently referred factors were identified.

### Procedures

The electronic version of the questionnaire was built on the Wenjuanxing platform (https://www.wjx.cn). WeChat publishing is a widely used mobile social platform that attracts a broad range of users in China. As of September 2015, WeChat had more than a billion created accounts and 650 million active users. Throughout the nation, the coverage of WeChat has more than 90% of smartphone users (18). It is considered a valid questionnaire if it is collected within 3 days, and the time for filling in the questionnaire is not < 15 min.

### Statistical analysis

Data were analyzed using the SPSS software package (Released 2017. IBM SPSS Statistics for Mac, version 25.0. Armonk, NY: IBM Corp.). The descriptive analysis uses the number and percentage of cases to describe the general data (such as gender, age, educational background, and professional title), makes an independent sample *t*-test on the scores of burnout and job stress of anesthesiologists of different gender, and makes one-way ANOVA on the situation of burnout and job stress of anesthesiologists of different age, professional title, education background, etc., among which, in one-way ANOVA, the variables with *P* < 0.15 were included in the multivariate analysis, the entry and removal criteria of multiple linear regression were 0.10 and 0.15, respectively. The multiple linear regression model was used to evaluate the independent correlation between the included variables, such as job stress and burnout. A two-tailed *P* < 0.05 was considered statistically significant.

## Results

The questionnaire was distributed and collected on April 2020. Of the 32 tertiary class A hospitals (there are 32 tertiary class A hospitals in three Northwest provinces) and 500 anesthesiologists and anesthesia residents, 32 hospitals and 336 individuals completed the survey (response rates 100% and 82.4%, respectively). The descriptive statistics are shown in Table 1.

**Table 1:** Descriptive statistics of the survey (*n* = 336).

**Project**	**Category**	**Number**	**Proportion (%)**
Gender			
	Male	157	46.73
	Female	179	53.27
Age (y)			
	< 25	6	1.79
	25–30	73	21.73
	31–35	79	23.51
	36–45	114	33.93
	>45	64	19.05
Education			
	Junior college and below	22	6.55
	Undergraduate	262	77.98
	Master	49	14.58
	Doctor	3	0.89
Title			
	Intern	3	0.89
	Resident physician	115	34.23
	Attending doctor	131	38.99
	Deputy chief physician	77	22.92
	Chief physician	10	2.98
Working life (y)			
	< 5	74	22.03
	5–10	79	23.51
	11–20	100	29.76
	21–30	66	19.64
	>30	17	5.06
Family financial situation			
	Rich	16	4.76
	General	227	67.56
	Income = expenditure	83	24.70
	Poor	10	2.98
Physical condition			
	Healthy	52	15.48
	Sub-health	251	74.70
	Suffer from disease	33	9.82
Type of hospital			
	Public teaching hospital	57	16.96
	Public specialized hospital	61	18.15
	Public general hospital	218	64.88
Anesthesiologist / OR			
	>3:1	14	4.17
	2.5–3:1	28	8.33
	2–2.5:1	53	15.77
	1–2:1	202	60.12
	< 1	39	11.61
Anesthesiologist / surgeon			
	< 0.1	53	15.77
	0.1–0.2	161	47.92
	0.21–0.3	73	21.73
	>0.3	49	14.58
Operation volume in recent two years			
	< 3,000	100	29.76
	3,000–6,000	80	23.81
	6,001–12,000	86	24.70
	12,001–20,000	25	7.44
	>20,000	48	14.29

Data are presented as frequency and percentage.

OR, operating room.

In Northwest China, the economy is relatively backward, the distribution of medical resources is unbalanced, and the level of medical and health services lags behind other regions (11).

The results of the burnout analysis showed that the scores of emotional exhaustion, depersonalization, and personal accomplishment were 20.23 ± 3.515, 17.98 ± 2.310, and 2.43 ± 0.670, respectively. 1. The emotional exhaustion of anesthesiologists with different working years and workloads was different, and the difference was statistically significant (*P* < 0.05); 2. The depersonalization of anesthesiologists with different ages, titles, working years, physical conditions, and workloads was different, and the difference was statistically significant (*P* < 0.05); and 3. The personal accomplishment of anesthesiologists with different physical conditions was different, and the difference was statistically significant (*P* < 0.05) (Table 2).

**Table 2:** Score of burnout ($\bar{\text{x}}$ ± s).

**Project**		**Emotion exhaustion**	**t/F**	***P* value**	**Depersonaliza-tion**	**t/F**	***P* value**	**Personal accomplishment**	**t/F**	***P* value**
All		20.23 ± 3.515			17.98 ± 2.310			2.43 ± 0.670		
Gender										
	Male	20.30 ± 3.603	0.357	0.721	17.90 ± 2.318	−0.624	0.533	2.36 ± 0.699	−1.837	0.067
	Female	20.16 ± 3.445			18.06 ± 2.307			2.50 ± 0.639		
Age (y)										
	< 25	21.17 ± 2.994	1.399	0.234	16.50 ± 1.871	19.849	< 0.001	2.67 ± 0.816	0.980	0.418
	25–30	19.96 ± 3.482			16.63 ± 1.837			2.36 ± 0.674		
	31–35	20.97 ± 3.158			17.41 ± 2.066^b^			2.52 ± 0.714		
	36–45	20.06 ± 3.561			18.48 ± 2.239^abc^			2.39 ± 0.645		
	>45	19.81 ± 3.866			19.48 ± 2.116^abcd^			2.48 ± 0.642		
Education										
	Junior college and below	19.86 ± 3.536	0.605	0.612	18.95 ± 2.572	1.829	0.142	2.36 ± 0.658	0.638	0.591
	Undergraduate	20.35 ± 3.465			17.98 ± 2.311			2.42 ± 0.683		
	Master	19.82 ± 3.828			17.57 ± 2.160^a^			2.55 ± 0.614		
	Doctor	18.67 ± 2.887			18.00 ± 1.000			2.33 ± 0.577		
Title										
	Resident physician	19.67 ± 3.786	1.324	0.261	16.67 ± 2.082	10.996	< 0.001	2.33 ± 1.155	0.316	0.867
	Attending doctor	20.41 ± 3.474			17.03 ± 2.178			2.41 ± 0.712		
	Deputy chief physician	20.31 ± 3.111			18.17 ± 2.160^b^			2.42 ± 0.667		
	Chief physician	20.14 ± 4.119			18.95 ± 2.265^bc^			2.48 ± 0.598		
	Resident physician	17.80 ± 3.676^bcd^			19.50 ± 1.900^b^			2.60 ± 0.699		
Working life (y)										
	< 5	19.76 ± 3.507	3.696	0.006	16.39 ± 1.893	23.336	< 0.001	2.42 ± 0.683	0.325	0.861
	5–10	21.10 ± 3.095^a^			17.39 ± 1.938^a^			2.48 ± 0.714		
	11–20	20.64 ± 3.518			18.59 ± 2.279^ab^			2.38 ± 0.663		
	21–30	19.11 ± 3.424^bc^			19.03 ± 1.969^ab^			2.47 ± 0.638		
	>30	20.12 ± 4.540			20.00 ± 2.151^abc^			2.47 ± 0.624		
Family financial situation										
	Rich	19.31 ± 4.254	1.236	0.296	18.06 ± 1.526	0.160	0.923	2.38 ± 0.719	0.582	0.627
	General	20.07 ± 3.598			17.94 ± 2.333			2.41 ± 0.662		
	Income = expenditure	20.80 ± 2.929			18.11 ± 2.225			2.52 ± 0.687		
	Poor	20.40 ± 4.624			17.70 ± 3.561			2.40 ± 0.699		
Physical condition										
	Healthy	19.33 ± 3.513	2.307	0.100	17.10 ± 2.320	6.463	0.002	2.52 ± 0.667	4.147	0.017
	Sub-health	20.33 ± 3.501			18.05 ± 2.270^a^			2.47 ± 0.647^a^		
	Suffer from disease	20.82 ± 3.486			18.85 ± 2.224^a^			2.19 ± 0.742^a^		
Type of hospital										
	Public teaching hospital	20.32 ± 3.704	0.236	0.918	17.70 ± 2.398	0.752	0.557	2.49 ± 0.658	0.343	0.849
	Public specialized hospital	20.22 ± 3.893			18.06 ± 2.402			2.50 ± 0.614		
	Public general hospital	20.29 ± 3.366			18.12 ± 2.282			2.41 ± 0.691		
Anesthes-iologists/OR										
	>3:1	20.14 ± 3.592	0.857	0.490	17.79 ± 1.888	1.380	0.240	2.14 ± 0.663	1.586	0.178
	2.5–3:1	19.89 ± 3.583			17.61 ± 1.812			2.29 ± 0.600		
	2–2.5:1	19.53 ± 3.577			17.72 ± 2.324			2.38 ± 0.657		
	1–2:1	20.37 ± 3.434			17.98 ± 2.366			2.47 ± 0.677		
	< 1	20.72 ± 3.790			18.72 ± 2.395^c^			2.56 ± 0.680^a^		
Anesthes-iologists / surgeon										
	< 0.1	21.17 ± 3.766	3.334	0.020	17.85 ± 2.348	1.090	0.354	2.62 ± 0.527	1.701	0.167
	0.1–0.2	20.40 ± 3.353			18.10 ± 2.200			2.39 ± 0.726^a^		
	0.21–0.3	19.26 ± 3.346^ab^			17.62 ± 2.202			2.40 ± 0.640^b^		
	>0.3	20.08 ± 3.752			18.29 ± 2.739			2.43 ± 0.645		
Operation volume in recent 2 years										
	< 3,000	19.85 ± 3.377	3.638	0.006	17.95 ± 2.208	4.345	0.002	2.45 ± 0.702	1.349	0.251
	3,000–6,000	20.29 ± 3.725			18.48 ± 2.624			2.49 ± 0.595		
	6,001–12,000	20.04 ± 3.337			17.69 ± 2.175b			2.34 ± 0.703		
	12,001–20,000	19.08 ± 3.475			16.56. ± 1.981^abc^			2.28 ± 0.737		
	>20,000	21.83 ± 3.379^abcd^			18.48 ± 2.010^d^			2.56 ± 0.616		

Data are presented as mean ± SD. The univariate analysis was performed using a *t*-test and ANOVA.

OR, operating room.

A *P* < 0.05 was regarded as statistically significant. Compared with the first group, ^a^*P* < 0.05. Compared with the second group, ^b^*P* < 0.05. Compared with the third group, ^c^*P* < 0.05. Compared with the fourth group, ^d^*P* < 0.05.

Analysis of job stress: anesthesiologists with different physical health statuses and workloads had different job stress, and the difference was statistically significant (*P* < 0.05) (Table 3).

**Table 3:** Score of job stress ($\bar{x}\pm$s).

**Project**		**CPSS**	**t/F**	***P* value**
All		7.51 ± 1.772		
Gender				
	Male	7.54 ± 1.712	0.314	0.754
	Female	7.48 ± 1.828		
Age (y)				
	< 25	7.83 ± 0.983	1.700	0.150
	25–30	7.07 ± 1.549		
	31–35	7.72 ± 1.825^b^		
	36–45	7.51 ± 1.806		
	>45	7.72 ± 1.890^b^		
Education				
	Junior college and below	7.68 ± 2.378	0.159	0.924
	Undergraduate	7.51 ± 1.699		
	Master	7.47 ± 1.916		
	Doctor	7.00 ± 1.000		
Title				
	Resident physician	7.00 ± 1.732	0.520	0.721
	Attending doctor	7.34 ± 1.696		
	Deputy chief physician	7.62 ± 1.778		
	Chief physician	7.61 ± 1.886		
	Resident physician	7.40 ± 1.838		
Working life (y)				
	< 5	7.07 ± 1.658	1.834	0.122
	5–10	7.82 ± 1.655		
	11–20	7.58 ± 1.865		
	21–30	7.50 ± 1.581		
	>30	7.59 ± 2.599		
Family financial situation				
	Rich	7.69 ± 2.522	0.186	0.906
	General	7.47 ± 1.791		
	Income = expenditure	7.54 ± 1.541		
	Poor	7.80 ± 1.989		
Physical condition				
	Healthy	6.96 ± 2.258	4.063	0.018
	Sub-health	7.55 ± 1.640^a^		
	Suffer from disease	8.03 ± 1.723^a^		
Type of hospital				
	Public teaching hospital	7.81 ± 1.620	0.652	0.625
	Public specialized hospital	7.46 ± 1.982		
	Public general hospital	7.48 ± 1.731		
Anesthesiologist/OR				
	>3:1	7.14 ± 2.349	1.728	0.143
	2.5–3:1	7.36 ± 1.660		
	2–2.5:1	7.28 ± 1.703		
	1–2:1	7.49 ± 1.779		
	< 1	8.15 ± 1.598^cd^		
Anesthesiologist / surgeon				
	< 0.1	7.72 ± 2.051	0.599	0.616
	0.1–0.2	7.55 ± 1.643		
	0.21–0.3	7.32 ± 1.763		
	>0.3	7.43 ± 1.893		
Operation volume in recent 2 years				
	< 3,000	7.34 ± 1.940	3.695	0.006
	3,000–6,000	7.71 ± 1.722		
	6,001–12,000	7.29 ± 1.542		
	12,001–20,000	6.88 ± 1.536^b^		
	>20,000	8.23 ± 1.777^acd^		

Data are presented as mean ± SD. The univariate analysis was performed using a t-test and ANOVA.

OR, operating room; CPSS, Chinese Perceived Stress Scale.

A *P* < 0.05 was regarded as statistically significant. Compared with the first group, ^a^*P* < 0.05. Compared with the second group, ^b^*P* < 0.05. Compared with the third group, ^c^*P* < 0.05. Compared with the fourth group, ^d^*P* < 0.05.

Correlation analysis of influencing factors of anesthesiologists' burnout: the longer the anesthesiologists' working life, the worse the health status and the more serious the depersonalization, and the difference was statistically significant (*P* < 0.05). The better the anesthesiologists' physical condition, the longer the working life and the higher the sense of personal accomplishment. The difference was statistically significant (*P* < 0.05) (Tables 4–6).

**Table 4:** Results of multiple linear regression of emotional exhaustion.

**Project**	**B**	**Std b**	**t**	***P* value**
Constant	18.548		14.346	< 0.001
Working life	−0.157	−0.052	−0.930	0.353
Anesthesiologist/OR	0.255	0.068	1.246	0.213
Anesthesiologist/surgeon	−0.392	−0.102	−1.869	0.062
Operation volume in recent 2 years	0.249	0.097	1.718	0.087

The entry and removal criteria of multiple linear regression were 0.10 and 0.15, respectively. For this model, R^2^ = 0.044, Adjusted R^2^ = 0.030, F = 3.052, and *P* = 0.010.

OR, operating room.

*P* < 0.05 was regarded as statistically significant.

**Table 5:** Results of multiple linear regression of depersonalization.

**Project**	**B**	**Std b**	**t**	***P* value**
Constant	13.967		18.034	< 0.001
Age	0.008	0.107	0.842	0.401
Education	−0.035	−0.007	−0.135	0.893
Title	−0.124	−0.046	−0.592	0.554
Working life	0.788	0.400	3.201	0.002
Physical condition	−0.608	−0.132	−2.693	0.007
Operation volume in recent 2 years	0.164	0.097	1.847	0.066

The entry and removal criteria of multiple linear regression were 0.10 and 0.15, respectively. For this model, R^2^ = 0.240, Adjusted R^2^ = 0.227, F = 17.354, and *P* < 0.010.

A *P* < 0.05 was regarded as statistically significant.

**Table 6:** Results of multiple linear regression of personal accomplishment.

**Project**	**b**	**Std b**	**t**	***P* value**
Constant	1.883		10.362	< 0.001
Gender	0.132	0.098	1.817	0.070
Physical condition	0.180	0.134	2.488	0.013
Working life	0.778	0.400	3,201	0.002

The entry and removal criteria of multiple linear regression were 0.10 and 0.15, respectively. For this model, R^2^ = 0.028, Adjusted R^2^ = 0.022, F = 4.807, and *P* = 0.009.

The *P* < 0.05 was regarded as statistically significant.

Correlation analysis of influencing factors of job stress of anesthesiologists: the better the physical health condition of anesthesiologists, the less the job pressure, and the difference was statistically significant (*P* < 0.05) (Table 7).

**Table 7:** Results of multiple linear regression of personal accomplishment job stress.

**Project**	**b**	**Std b**	**t**	***P* value**
Constant	5.008		7.943	< 0.001
Age	0.280	0.171	1.294	0.196
Working life	−0.150	−0.099	−0.742	0.459
Physical condition	−0.477	−0.135	−2.471	0.014
Anesthesiologist/OR	0.180	0.095	1.749	0.081
Operation volume in recent 2 years	0.133	0.102	1.843	0.066

The entry and removal criteria of multiple linear regression were 0.10 and 0.15, respectively. For this model, R^2^ = 0.049, Adjusted R^2^ = 0.034, F = 3.379, and *P* = 0.005.

OR, operating room.

*P* < 0.05 was regarded as statistically significant.

## Discussion

This study assesses both burnout and job stress among anesthesiologists and provides a good understanding of the current psychological status of anesthesiologists among all colleagues and related personnel in the anesthesia industry. Using Northwest China as the background of the study is relevant and can cause the relevant departments to care for anesthesiologists in Northwest China and incline resources. However, this study also has limitations. For example, the sample size was limited. We distributed 500 questionnaires, yet only 406 were returned, of which only 336 were valid, which may have led to selection bias in the study results. In further research studies in the future, we can expand the sample size, keep in touch with the study participants after distributing the questionnaire, and supervise them to complete the questionnaire to reduce selective bias.

The medical and health industry is a profession with high requirements and a low sense of control, and a profession with a high incidence of burnout (19). At present, there are 76,000 anesthesiologists in China. The number of anesthesiologists per 10,000 people in China is ~0.4, compared with three in the United States and 2.8 in the United Kingdom. According to British and American standards, China should have 310,000 anesthesiologists. In fact, the total number of anesthesiologists in China is < 100,000 and also less than one-third of the standard configuration, and the gap is 200,000 (20). At the same time, China's surgery volume accounts for approximately 10% of the global surgery volume, and it is still growing at a rate of 10% (21). In recent years, the doctor–patient relationship has become increasingly tense, coupled with factors such as the income, promotion, and physical condition of anesthesiologists, which make the environment of anesthesiologists worse, thus, job stress and burnout are increasing day by day (22).

As for emotional exhaustion, the phenomenon of emotional exhaustion with working years of 5–10 years is serious. Most anesthesiologists who have worked for 5–10 years have accumulated professional knowledge and skills and can play an independent role at work. In hospitals at all levels, they are the main force of their department. Therefore, their emotional exhaustion is severe, which is consistent with the research results of Filippo (23). With the increase in working years, the phenomenon of emotional exhaustion began to ease. This may be explained by the fact that, with the continuous increase of working years, their medical experience continues to be accumulated, and technical literacy is more and more suitable for the needs of the post, and then, the burnout will reduce. The higher the annual operation volume is, the more serious the emotional exhaustion is. The increase in workload will directly lead to the excessive fatigue of anesthesiologists. Excessive fatigue not only affects the physical and mental health of anesthesiologists but also causes great potential harm to patients. In the study of Gordon (24), it was found that as many as 50% of the anesthesiologists interviewed admitted that they had medical errors when they were tired.

With the increase in age, working years, and professional titles of anesthesiologists, their depersonalization is more and more serious. With the increasing age and working years of an anesthesiologist, the accumulated fatigue, work pressure, dissatisfaction, and sub-health will reach a peak, which makes the phenomenon of depersonalization more serious. The phenomenon is manifested in negative work and indifference to colleagues. From the correlation analysis, it is found that the length of working years is positively related to the occurrence of depersonalization, while the health status is negatively related to the occurrence of depersonalization, and the difference is statistically significant. Therefore, the relevant departments should pay attention to the physical and mental health of anesthesiologists, which will help to delay depersonalization and improve burnout (25).

In Northwest China, the better an anesthesiologist's health is, the higher his accomplishment will be. In Northwest China, the economy is underdeveloped, and the medical level is relatively backward. People are still in the dilemma of difficult and expensive medical treatment (26). Novel coronavirus pneumonia (COVID-19) has been prevalent worldwide in 2020, which has caused millions of people to die. The number of deaths has reached 60,000, and the number of infections and deaths is rising (27, 28). This makes us more convinced that nothing matters except for a healthy body. A healthy body can bring more sense of personal achievement to anesthesiologists in Northwest China. There was a positive correlation between personal accomplishment, physical health, and working years, and the difference was statistically significant. With the accumulation of working years, the academic level, experience and technology, and salary and income of anesthesiologists will reach a new level, and the sense of personal accomplishment will naturally increase. Coupled with a healthy body, a great sense of personal accomplishment can often be obtained, reducing the occurrence of burnout.

In total, 88.1% of anesthesiologists in this questionnaire had high job stress, among whom the greater the workload, the worse the physical health, and the higher the job stress of anesthesiologists, and the difference was statistically significant. In the correlation analysis, job stress was negatively correlated with physical health, and the difference was statistically significant. The aforementioned shortage of anesthesiologists in China and the increasing workload lead to the physical and mental fatigue of medical staff and the sudden rise of pressure (29). In addition, the sudden death rate of anesthesiologists is increasing year by year. According to the researchers such as Shan, the proportion of sudden death of anesthesiologists in China is the highest, reaching 26% (30). As for the reasons of excessive workload, worry about medical litigation, sleep deprivation, and interpersonal relationship processing, anesthesiologists will often be in a certain degree of physiological or psychological disease states (31), and anesthesiologists in a sick state will naturally feel the job stress increase at work. Therefore, the healthy body and mind of an anesthesiologist are the premise and guarantee of his normal work.

In conclusion, burnout and high job pressure are common among anesthesiologists in Northwest China. The increasing workload, unfair promotion system, and long-term sub-health lead to a decline in their physical health. In terms of anesthesiologists themselves, they should improve their psychological quality, maintain their mental health, constantly enrich and improve their professional level, and improve the communication ability between doctors and patients (32). For the department leaders and the health administrative departments, we should first reduce the risk of occupational exposure of anesthesiologists, then adjust the overall allocation of resources in the hospital, pay attention to the improvement and renewal of medical equipment, and more importantly, ensure the fairness and impartiality of the salary and promotion system, strengthen the overall input and construction of the anesthesiology department, and strive to establish contact with the international community soon (33). Only when we pay attention to the problems of anesthesiologists, we can guarantee the safety and service quality of patients in the northwest region and even in the whole country, and anesthesiologists in China can make great progress (34).

## Data availability statement

The raw data supporting the conclusions of this article will be made available by the authors, without undue reservation.

## Ethics statement

The studies involving human participants were reviewed and approved by the Research Ethics Committee, Xi'an Honghui Hospital Affiliated to Xi'an Jiaotong University. The patients/participants provided their written informed consent to participate in this study.

## Author contributions

J-mY: conceptualization. GY: writing—original draft preparation. L-yP: writing—reviewing and editing. B-hD: methodology. X-lF: software. ZQ: visualization. All authors have read and agreed to the published version of the manuscript.
